# Supplementary material for: Intracerebral hemorrhage induces monocyte TNF signaling that is suppressed by Siponimod (BAF312): a single-cell transcriptomics study in patients
Source: medRxiv. 2026 Jan 27:2026.01.22.26344292. Preprint. [Version 1] doi: 10.64898/2026.01.22.26344292 (PMC12870585; doi:10.64898/2026.01.22.26344292)
Supplement: Supplement 12 [file NIHPP2026.01.22.26344292v1-supplement-12.pdf]

medRxiv preprint doi: https://doi.org/10.64898/2026.01.22.26344292; this version posted January 27, 2026. The copyright holder for this preprint (which was not certified by peer review) is the author/funder, who has granted medRxiv a license to display the preprint in perpetuity.
It is made available under a CC-BY 4.0 International license .

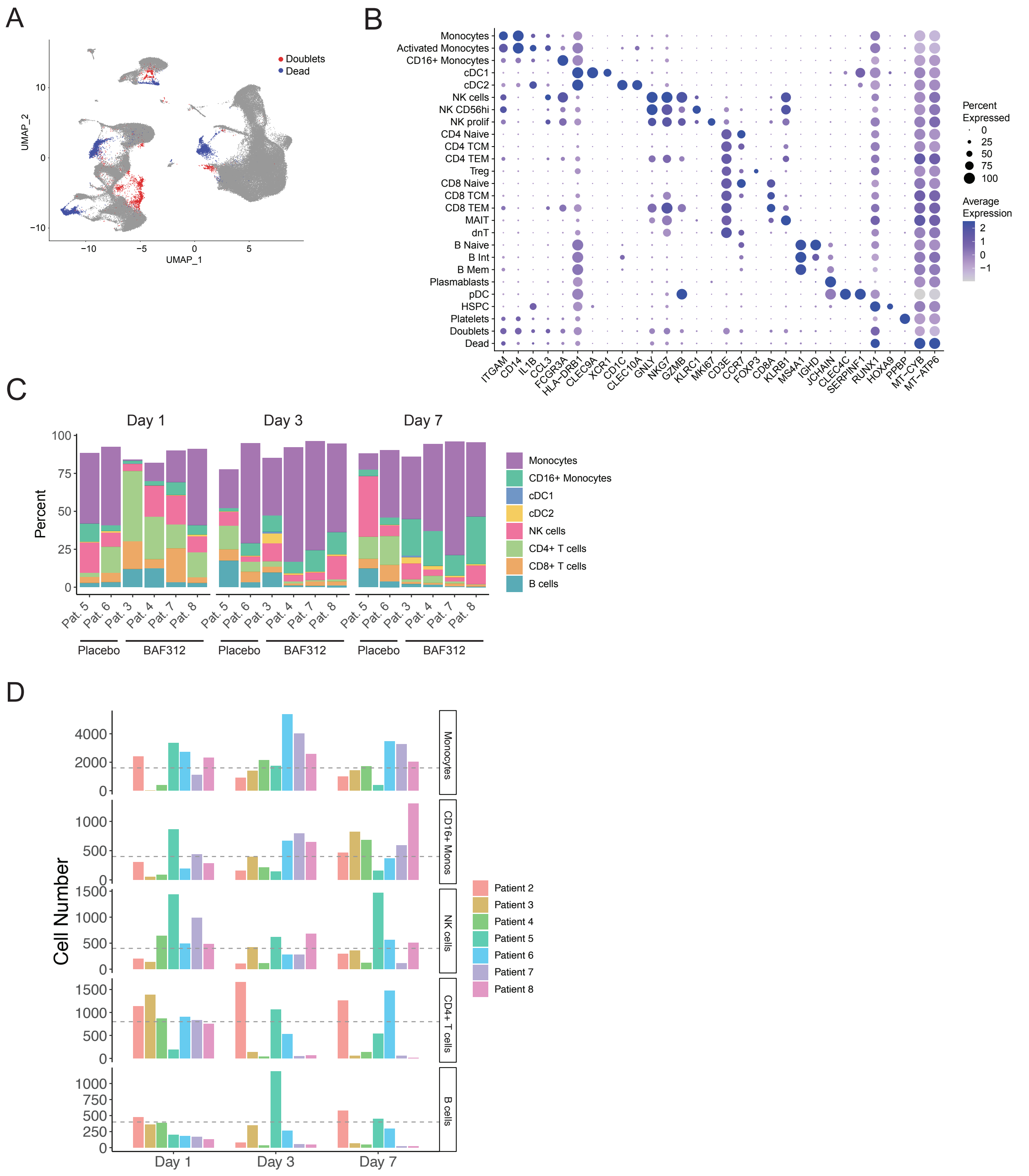

Supp Fig 1. Clustering and downsampling. (A) UMAP including doublets and dead cells that were removed before downstream analysis. (B) Expression dotplot of select genes that help identify the indicated subclusters of cells. (C) Proportions of cells found in select major cell lineages, in

medRxiv preprint doi: https://doi.org/10.64898/2026.01.22.26344292; this version posted January 27, 2026. The copyright holder for this preprint (which was not certified by peer review) is the author/funder, who has granted medRxiv a license to display the preprint in perpetuity.
It is made available under a CC-BY 4.0 International license .

each patient, at each timepoint. Proportions do not add to 100% because minor lineages are not represented. (D) Cell numbers collected per lineage, per sample. Dashed lines indicate the number of cells to which each cell type was downsampled.

medRxiv preprint doi: https://doi.org/10.64898/2026.01.22.26344292; this version posted January 27, 2026. The copyright holder for this preprint (which was not certified by peer review) is the author/funder, who has granted medRxiv a license to display the preprint in perpetuity.
It is made available under a CC-BY 4.0 International license .

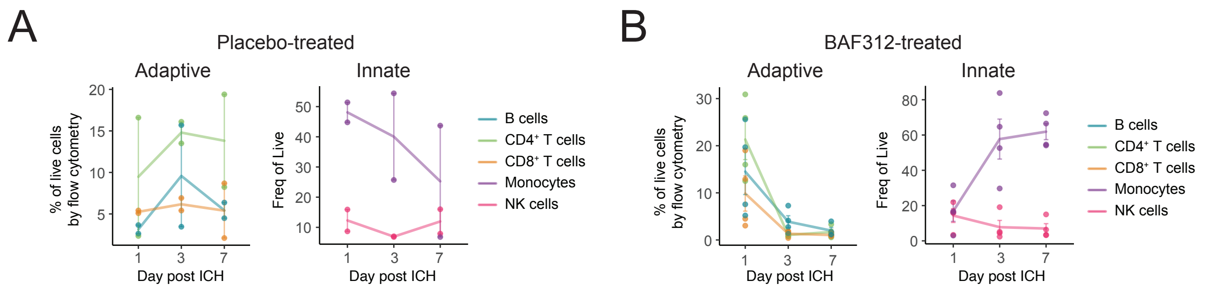

Supp Fig 2. Population frequency changes after ICH by flow cytometry. (A-B) Cell proportions in placebo-treated patients (A) and BAF312-treated patients (B), measured by flow cytometry. Adaptive immune populations are shown on the left and innate immune populations are shown on the right.

medRxiv preprint doi: https://doi.org/10.64898/2026.01.22.26344292; this version posted January 27, 2026. The copyright holder for this preprint (which was not certified by peer review) is the author/funder, who has granted medRxiv a license to display the preprint in perpetuity.
It is made available under a CC-BY 4.0 International license .

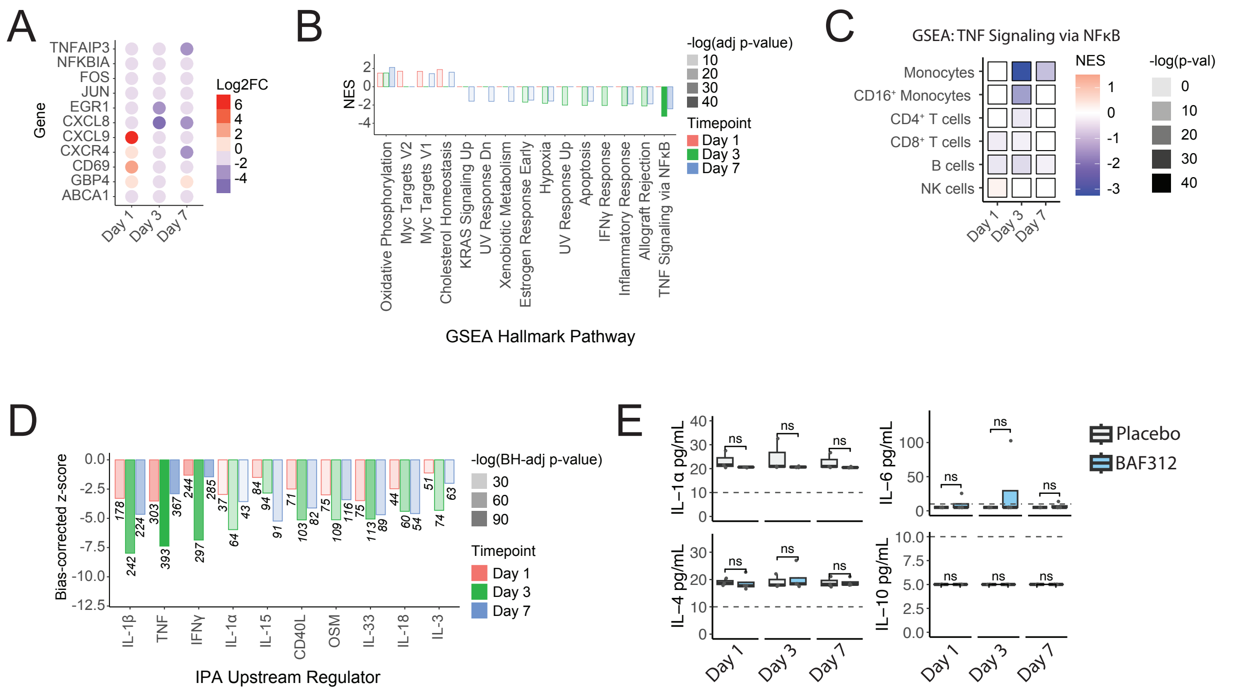

Supp Fig 3. BAF312 decreased inflammatory response compared to placebo. (A-D) Samples were pseudobulked by cell type and fit to a linear model in which gene expression is normalized for ICH score. (A) Differential expression of select inflammatory genes in monocytes from BAF312-treated vs placebo-treated patients. (B) GSEA of monocytes from BAF312-treated vs placebo-treated patients. (C) GSEA of different cell lineages in BAF312-treated vs placebo-treated patients, showing only results for MSigDB Hallmark Pathways TNFA_SIGNALING_VIA_NFKB pathway. (D) Enrichment of cytokine signaling pathways in BAF312-treated vs placebo-treated patients, measured by Ingenuity Pathways Analysis Upstream Regulators Analysis. (E) Concentration of cytokines in patient plasma, measured by Cytometric Bead Array. Dashed lines indicate lower limit of detection.

medRxiv preprint doi: https://doi.org/10.64898/2026.01.22.26344292; this version posted January 27, 2026. The copyright holder for this preprint (which was not certified by peer review) is the author/funder, who has granted medRxiv a license to display the preprint in perpetuity.
It is made available under a CC-BY 4.0 International license .

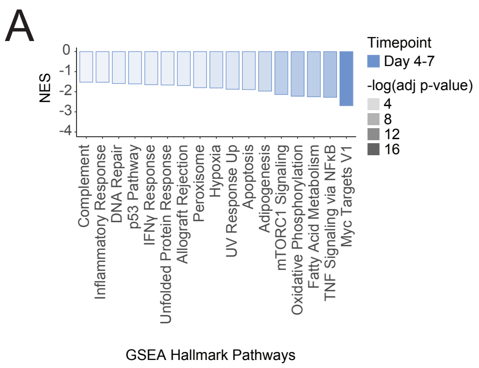

Supp Fig 4. Association of outcome with immune response in MISTIE III study. GSEA of monocytes isolated from the peripheral blood of ICH patients, comparing mRS of 0-3 vs 4-6 at 1 year post ICH, from Askenase, et al 2021.

medRxiv preprint doi: https://doi.org/10.64898/2026.01.22.26344292; this version posted January 27, 2026. The copyright holder for this preprint (which was not certified by peer review) is the author/funder, who has granted medRxiv a license to display the preprint in perpetuity.
It is made available under a CC-BY 4.0 International license .

SUPPLEMENTAL TABLE LEGENDS

Supp Table 1. Sequencing metrics. Sequencing quality metrics as reported by Cell Ranger v7.1.0

Supp Table 2. Patient demographics and clinical characteristics. Patient demographics, medical history, central reading data, and clinical outcome measurements.

Supp Table 3. DEG of Placebo-treated patients – comparisons by timepoint. Differential gene expression, performed using the FindMarkers() function as described in Methods. DEGs were calculated for each of the 6 main lineages identified. Three comparisons were made for each population in placebo-treated patients: Day 3 vs Day 1, Day 7 vs Day 3, and Day 7 vs Day 1.

Supp Table 4. GSEA of Placebo-treated patients – comparisons by timepoint. GSEA was performed using the fgsea() function of the fgsea package in R. Input genes were the DEGs shown in Supp Table 3, ranked by log_2(fold-change).

Supp Table 5. DEG of BAF312-treated patients – comparisons by timepoint. Differential gene expression, performed using the FindMarkers() function as described in Methods. DEGs were calculated for each of the 6 main lineages identified. Three comparisons were made for each population in BAF312-treated patients: Day 3 vs Day 1, Day 7 vs Day 3, and Day 7 vs Day 1.

medRxiv preprint doi: https://doi.org/10.64898/2026.01.22.26344292; this version posted January 27, 2026. The copyright holder for this preprint (which was not certified by peer review) is the author/funder, who has granted medRxiv a license to display the preprint in perpetuity.
It is made available under a CC-BY 4.0 International license .

Supp Table 6. GSEA of BAF312-treated patients – comparisons by timepoint. GSEA was performed using the fgsea() function of the fgsea package in R. Input genes were the DEGs shown in Supp Table 5, ranked by log_2(fold-change).

Supp Table 7. DEG of placebo-treated vs BAF312-treated patients. Differential gene expression, performed using the FindMarkers() function as described in Methods. DEGs were calculated for each of the 6 main lineages identified. Three comparisons were made for each population: placebo-treated vs BAF312-treated at Day 1, Day 3, and Day 7.

Supp Table 8. GSEA of placebo-treated vs BAF312-treated patients. GSEA was performed using the fgsea() function of the fgsea package in R. Input genes were the DEGs shown in Supp Table 7, ranked by log_2(fold-change).

Supp Table 9. DEG of pooled patients by clinical characteristics. Differential gene expression, performed using the FindMarkers() function as described in Methods. DEGs were calculated for each of the 6 main lineages identified. Patients were bifurcated into groups based on clinical measures of ICH severity and outcome and DEGs were identified that distinguish these groups at Day 1, Day 3, and Day 7.

medRxiv preprint doi: https://doi.org/10.64898/2026.01.22.26344292; this version posted January 27, 2026. The copyright holder for this preprint (which was not certified by peer review) is the author/funder, who has granted medRxiv a license to display the preprint in perpetuity.
It is made available under a CC-BY 4.0 International license .

Supp Table 10. GSEA of pooled patients by clinical characteristics. GSEA was performed using the fgsea() function of the fgsea package in R. Input genes were the DEGs shown in Supp Table 9, ranked by $\log_2$(fold-change).

Supp Table 11. Supporting Data Values. Raw data values are included for all plots displaying non-transcriptome data.
